# Efficiency of Erbium-Doped Yttrium Aluminum Garnet Laser in Debonding Cemented Glass Fiber Posts: an *in vitro* Study

**DOI:** 10.30476/DENTJODS.2020.83933.1062

**Published:** 2021-03

**Authors:** Amir Hossein Zamanian, Seyed Mohammad Reza Hakimaneh, Seyed Masoud Mojahedi, Farnaz Taghavi Damghani, Fatemesadat Shayegh, Sayed Shojaedin Shayegh

**Affiliations:** 1 Postgraduate Student, Dept. of Prosthodontics, Faculty of Dentistry, Shahed University, Tehran, Iran; 2 Dept. of Prosthodontics, Faculty of Dentistry, Shahed University, Tehran, Iran; 3 Laser Application in Medical Sciences Research Center, Shahid Beheshti University of Medical Sciences, Tehran, Iran; 4 Dental Student, Faculty of Dentistry, Shahed University, Tehran, Iran

**Keywords:** Laser, Resin Cements, Root Canal Therapy, Er YAG, Bond Strength

## Abstract

**Statement of the Problem:**

The efficacy of erbium-doped yttrium aluminum garnet (Er; YAG) laser on the debonding properties of certain post materials has remained largely unexplored.

**Purpose:**

This study aimed to investigate the effect of Er; YAG laser irradiation on debonding of cemented glass fiber posts in root canal treated teeth.

**Materials and Method:**

In this *in vitro* study, forty root canal treated mandibular premolar teeth were used in this study. Glass fiber posts were bonded using Panavia F 2.0 cement in the root canal space, and samples were divided into two groups. In the test group, samples were exposed to laser radiation of 7W, 350mJ, frequency of 20Hz and discontinued washing spray. In the control group, samples were left untouched. In each group, samples were sectioned into 1.5mm thick slices from the coronal, middle, and apical thirds of the root (N=120). Tensile bond strengths were evaluated using the push-out test and the failure patterns were evaluated using scanning electron microscopy (SEM). To compare the laser and non-laser groups at each location, independent sample t test was applied, and to compare bond strength between the locations in each group, one-way ANOVA and Tukey’s HSD post hoc was applied.

**Results:**

A significant difference in tensile strength was observed between the laser-irradiated group and control group; tensile bond strength was much higher in the control group (*p*<0.001). The highest frequency of fractures was observed at the cement-dentin interface. Given the used parameters, complete debonding was not achieved in the laser-irradiated group.

**Conclusion:**

Laser radiation reduced the bond strength of glass fiber posts to resin cement without complete debonding.

## Introduction

Teeth with insufficient residual coronal structure, caused by caries lesion, fracture, or extensive cavity preparation can be treated with various endodontic posts. One of the main functions of endodontic posts is to provide retention and stability for favorable coronal restoration [ 1 ]. Disadvantages associated with metal posts have led to the development of different esthetic posts among which prefabricated posts have gained popularity due to their adhesive capacity and ability to form a gap-free single unit. Prefabricated posts can be made of zirconia, glass, quartz or polyethylene fibers [ 2 ]. To select an appropriate material for prefabricated posts, fracture resistance should be considered as an important physical property. Carbon and glass fiber posts are favorable in this manner [ 3 ]. Satisfactory survival rates have been reported in teeth that have been restored with fiber reinforced composite (FRC), posts made of quartz or glass fibers embedded in a matrix of epoxy or methacrylate resin [ 4 ].

However, considerations for appropriate post selection should not be limited to fracture resistance and must include efficiency of post removal. In several incidences, post removal and endodontic retreatment is required due to unsatisfactory post length, diameter, or apical seal [ 5 ]. Different techniques for post removal are selected based on post material and device availability, including burs to drill, ultrasonic vibration, and solvents used with endodontic files [ 6 ]. These methods can be relatively challenging, increase the risk of fracture [ 7 ], and cause pain and discomfort to the patient [ 8 ].

Since their introduction in the 1990s, lasers have revolutionized modern dental practice. Lasers can be applied for several purposes, such as diagnostic applications (caries detection), tooth whitening, resin curing, and cavity preparation [ 9 ]. Erbium: yttrium aluminum garnet (Er: YAG) laser, is readily used for cutting enamel, dentin, and gingival depigmentation procedures [ 10 ], due to its bactericidal effects and ability to cause minimal pain [ 11 ]. Moreover, Er: YAG has been used in the removal of glass ceramic brackets and porcelain veneers [ 12 - 14 ]. Namely, Oztoprak *et al*. [ 12 ] reported the effectiveness of Er: YAG laser on thermal softening and degradation of adhesives, resulting in debonding of ceramic brackets. Moreover, Morford *et al*. [ 14 ] conducted a study on IPS Empress and IPS e.max porcelain veneers, concluding that the application of Er: YAG laser can be effective in debonding and preserving tooth structure. Despite these developments, the debonding properties of Er: YAG laser on different post materials remains largely unexplored.

Therefore, in this study, the effects of Er: YAG laser on debonding cemented glass fiber posts in endodontically treated teeth is investigated. The null hypothesis was that laser irradiation could not reduce the bond strength of glass fiber posts.

## Materials and Method

A minimum sample number of 36 (power of 80%) was calculated. However, for higher accuracy, 40 samples were recruited and consented for the study. Samples included single-rooted mandibular premolars with the same length, extracted due to periodontal, prosthetic or orthodontic reasons, which were divided into two groups (20 in each group).

For disinfection, each tooth was stored in 5% chloramine T (Mina Tajhiz-co, Tehran, Iran) for 48 hours, before placing in distilled water, which was changed every day until examination. Soft tissue and dental plaques were removed from the root surface using periodontal scaler. Two radiographs were taken to assess the anatomic structure. Any tooth with internal/external resorption, two or more roots or canals, calcification, fracture lines and cracks detected by stereomicroscope was excluded from the study. Each tooth was sectioned from the crown with a water-cooled diamond disk (Buehler, Lake Bluff, Illinois, USA) to reach a standard root height of 15 mm.

Root canal therapy was performed for each tooth using the step back method, which then obturated using a gutta-percha (DiaDent, DiaDent Group International, Chongju, Korea) and sealer (AH Plus; Dentsply De Trey GmbH, Konstanz, Germany), applying the cold lateral condensation method [ 15 ]. The access hole was filled with temporary restorative material (Cavisol, Gpl Chai co., Alborz, Iran) and teeth were placed in distilled water.

After 24 hours, the gutta-percha was removed using Gates Glidden drills (Dentsply Maillefer, Ballaigues, Switzerland), leaving a 5mm of the gutta-percha at the end of the canal. Fiber posts (Whitepost DC 2; FGM Produtos Odontologicos, Joinville, Brazil) with a height of 20mm, end diameter of 1.8mm and tip diameter of 1.05mm were used in this study. For fiber post placement, the root canals were prepared using drills according to the manufacturer’s instructions. The depth of 10 mm was prepared for the post space.

The prepared post space then rinsed with 2% chlorhexidine (Drogal Farmácia de Manipulação Ltda., Piracicaba, Brazil), before drying with paper points (DiaDent, DiaDent Group International, Chongju, Korea). Excess length of the fiber posts were cut outside the canal (by drilling), and then the fibers surface was dipped in ProsilSilane (FGM, Joinville, SC, Brazil) and dried. The fiber posts were cemented using Panavia F2.0 cement (Kuraray Inc., NY, USA) according to the manufacturer’s instructions. Er:YAG laser (wave length of 2,940 nm, 20 Hz) was used in the contact mode for 60 seconds. The selected parameters were based on Hoteit *et al*. [ 16 ] to prevent extreme heat generation and decrease crack formation. A pilot study was conducted to determine the best parameters for laser irradiation. Six extra samples were randomly selected for the pilot study. Table 1 presents the tested parameters. According to the results of the pilot study, the best effects were obtained using 7 W, interrupted pulse duration and discontinued rinsing spray. For the main study, 40 samples were divided into two groups by simple randomization method (using a hexagonal dice). The first group received no laser and the second group was irradiated with the laser. The laser tip was placed on the coronal part, exactly on the fiber post, at the cut site.

**Table 1:** Laser parameters used for the pilot study

Cooling	Pulse duration	Potency
Continuous water spray(5mL/min)	Continuous	4 Watt
Continuous water spray(5mL/min)	Continuous	6 Watt
Continuous water spray(5mL/min)	Continuous	7 Watt
Interrupted water spray(5mL/min)	Interrupted	4 Watt
Interrupted water spray(5mL/min)	Interrupted	6 Watt
Interrupted water spray(5mL/min)	Interrupted	7 Watt

**Figure 1:**
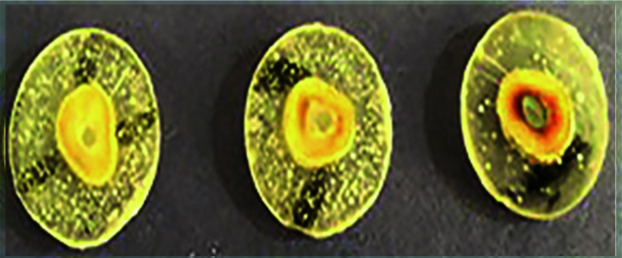
Samples cut from 1/3 coronal, 1/3 middle, and 1/3 apical sections

Push-out test was used to evaluate tensile resistance. After laser irradiation, 40 teeth (20 premolars in each group) were sectioned under a microscope (Leica S8 APO; Leica Microsystems Inc., IL, USA) with a disk (Buehler, Lake Bluff, Illinois, USA) to obtain 1.5 mm thick slices (Figure 1) according to the penetration depth of Er:YAG laser [ 17 ] from the coronal, middle and apical thirds of the root (120 slices in total). The diameter of each cut was measured with a digital caliper (Mitutoyo digital caliper; Mitutoyo, IL, USA). For the push-out test, the cut samples were placed on the universal testing machine (Instron, MA, USA), with an empty space under the post. A load with a 1mm/min cross head speed [ 18 ] was applied to the post and the load at which the fracture occurred was recorded. Tensile strength was calculated by dividing the load of fracture (N) by bonding area (A), and the value was reported in MPa. 

The bonding area was calculated according to the equation [ 18 ]: [(R+r)*h^2^ + (R−r)^2^]

where R is the coronal diameter of the post, r is the apical diameter of the post and h is the slice thickness in mm.

Five samples from each group were randomly selected, which were prepared for scanning electron microscopy (SEM) analysis. Samples were coated with gold and evaluated using SEM (Zeiss Evo 50; Carl Zeiss, Oberkochen, Germany) at a magnification of ×100. Finally, the fracture mode was reported as cohesive fracture in post, cements, dentin, adhesive failure between cement and post, adhesive failure between cement and dentin, and mixed.

Statistical analysis

The effects of laser irradiation and location on bond strength were evaluated using two-way ANOVA. As the interaction effect was significant, subgroup analysis was applied. Independent sample t test was used to compare lasered and non-lasered groups in each location and one -way ANOVA and Tukey’s HSD post hoc test were used to compare the strength between locations in each group.

## Results

**Figure 2:**
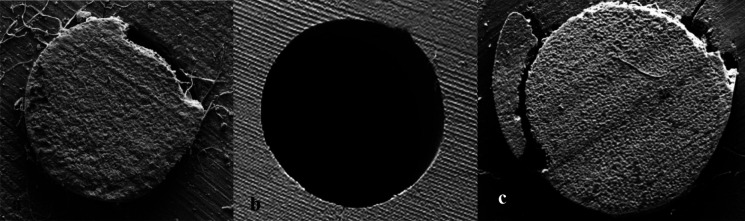
SEM images showing fracture sites. **a:** Cohesive fracture in post, **b:** Adhesive failure between cement and dentin, **c:** The mixed fracture.

In this study, 20 teeth in each group and 3 cuts on each tooth resulted in 120 samples. Tensile strength measurements are presented in Table 2. Statistically significant difference in push-out bond strength was noted between the groups. Tensile strengths at different sections were higher in the control group than the laser-irradiated group (*p*< 0.001). Within group comparison analysis, significant differences in the control group were noted; the highest tensile strength was related to the coronal section (10.303 MPa) and the lowest was belonged to the apical section (6.842 MPa). There was a significant difference between coronal and middle sections (*p*=0.022) and between middle and apical sections (*p*<0.001; Table 2). In the laser-irradiated group, the highest tensile strength was belonged to the coronal section (4.023MPa) and the lowest to the apical section (3.308MPa), which were not significantly different (*p*= 0.09). Also no significant differences were noted between the coronal and middle (*p*= 0.680) sections and the middle and apical sections (*p*= 0.399), as shown in Table 2. SEM images of the fractures in each site are shown in Figure 2.

**Table 2:** Comparison of tension resistance of glass fiber posts between the two study groups

Study group	Studied sections	Mean±SD	Minimum- Maximum	Comparison with	Mean difference	*p* Value	Lower limit	Upper limit
Laser irradiated group	1/3 coronal	4.023±0.948	1.222-6.109	Compared with middle	0.280150	0.680	−0.52299	1.08329
				Compared with apical	0.715200	0.090	−0.08794	1.51834
	1/3 middle	3.743±1.119	1.127-5.218	Compared with coronal	−0.280150	0.680	−1.08329	0.52299
				Compared with apical	0.435050	0.399	0.36809	1.23819
	1/3 apical	3.308±1.090	1.056-5.157	Compared with coronal	−0.715200	0.090	−1.51834	0.08794
				Compared with middle	−0.435050	0.399	−1.23819	0.36809
Control group	1/3 coronal	10.303±1.139	7.805-12.259	Compared with middle	0.895600*	0.022	0.11045	1.68075
				Compared with apical	3.461650*	<0.001	2.67650	4.24680
	1/3 middle	9.408±0.972	7.231-11.647	Compared with coronal	0.895600*	0.022	−1.68075	−0.11045
				Compared with apical	2.566050*	<0.001	1.78090	3.35120
	1/3 apical	6.842±0.974	4.029-8.230	Compared with middle	−3.461650*	<0.001	−4.24680	−2.67650
				Compared with coronal	−2.566050*	<0.001	−3.35120	−1.78090

* Significant at <0.05, according to the results of *t*-test

The comparison of distribution of fractures at the five sites between the laser-irradiated and the control group showed that the highest number of fractures occurred at cement-dentin interface. Three fractures were occurred in the cement-dentin interface. One fracture was mixed and one was cohesive in the post.

## Discussion

In the present study, we evaluated the effect of Er: YAG laser on the debonding of glass fiber posts cemented using resin cement. The findings showed that Er: YAG laser effectively reduced bond strength of the glass fiber post, rejecting the null hypothesis.

The pilot study showed that maximum bond strength reduction was obtained by employing the following parameters: 7 W, 350 mJ, pulse length of1 min duration, frequency of 20 Hz, and discontinued rinsing spray. In the main study, although complete debonding was not achieved, the results showed significant bond strength reduction in the laser-irradiated group. Moreover, the push-out test results showed a significantly higher tensile resistance in the control group than the laser-irradiated group.

To the author’s knowledge, just one study has been reported the efficacy of Er: YAG laser on debonding glass fiber posts using resin cement [ 19 ]. This study reported that the advantage of using laser instead of ultrasonic to retrieve glass fiber posts is causing lower temperature at the root surface and the post removal can be 5 times faster by irradiation of Er:YAG [ 19 ]. These results are in accordance with the results of the present study. Different studies have suggested that Er: YAG laser is efficient in removing composite restorations and debonding ceramic brackets [ 12 - 14 ]. In a bovine study, irradiation of Er: YAG (4.2W for 9 seconds) using the scanning method successfully enabled debonding polycrystalline ceramic brackets with lower shear bond strengths and higher adhesive remnant index [ 12 ]. Moreover, Mondethu *et al*. [ 13 ] investigated the effect of Er: YAG (600 mJ per pulse, 800 μs duration) on debonding of ceramic orthodontic brackets and reported successful debonding in 95% of the samples without damaging the enamel.

Differences between the results of the present study and the above-mentioned studies can be due to the study design; explicitly, this study was *in vitro*, while both the above-mentioned studies were investigated in vivo [ 12 - 14 ]. Additionally, laser parameters vary greatly among the different studies, which could justify different results [ 20 ].

Er: YAG laser is effective in debonding porcelain veneers [ 14 ] and ceramic restorations [ 21 ] with thermal and photo ablation of the resin cement [ 21 ]. In photo-induced thermo-mechanical ablation, water absorbs energy as an intermediate component. In composite resin ablation, the explosive vaporization followed by hydrodynamic ejection (the pressure of the water vapor and melted organic components of resin) is sufficient for debonding of restorations, and by selecting a laser with long pulse duration, an ablative effect on the cement can be demonstrated without destroying the tooth structure [ 21 ].

In this study, the highest tensile resistance belonged to the coronal section of the control group (10.303 MPa) and the lowest tensile strength belonged to the apical section of the laser-irradiated group (3.308 MPa). However, the difference between root sections in laser group was not significant. Distance from the light source [ 22 ], translucency of the fiber post [ 23 ] and limitations in the flow of the cement to the apical section of the canal are factors that could affect the degree of polymerization [ 24 ]. Onay *et al*. [ 24 ] conducted a study on glass fiber posts cemented with different luting agents and showed a significant reduction in bond strength from the cervical to the apical third. However, in the laser group, the reduction of bond strength in coronal sections could occur and resulted in similar bond strength with middle and apical sections.

Other studies have shown the debonding effect of Nd: YAG laser on orthodontic brackets and metal posts [ 25 - 26 ]. Takashina *et al*. [ 26 ] investigated the use of Nd: YAG laser in debonding dowels made of a variety of metals, zinc phosphate, glass ionomer, and resin cements. They reported a minimum of 110 seconds and a maximum of 812 seconds for complete dowel debonding, and significantly faster debonding occurred in silver alloy than other metals. Noteworthy, the authors applied discontinued laser irradiation upon complete debonding. However, in the present study, the samples were irradiated for one minute. A longer duration may have possibly resulted in complete debonding in the above mentioned study. Such findings suggest that the length of irradiation may play an important role in debonding outcomes. However, by increasing the time and power of laser irradiation, the heat would be increased and could damage the tooth and periodontal tissues that should be taken into account in adjusting the laser parameters.

In the present study, post spaces were pretreated with chlorhexidine, which have been reported to increase bonding strength [ 27 - 28 ]. Different factors can play a role in the debonding efficacy of lasers, such as the type of rinsing material [ 29 - 30 ], resin cement [ 21 , 31 ] and root canal sealer [ 32 ] used. Aging can also affect bonding strength [ 33 ], although this was not evaluated in this study.

According to the fracture site, the highest fracture frequency was observed between the resin-dentin interfaces in both groups. This finding could reveal that the bond strength in the interface of cement-dentin is weaker than the cement-post interface. But, according to the low sample size of the present study, this finding should be confirmed by future studies. Similarly, on a study conducted on fiber-reinforced composite post systems, Kececi *et al*. [ 34 ] observed fewer cohesive failures in cement or posts than adhesive failures between dentin and cement.

Nevertheless, there are some limitations in the present study. The influence of aging in this *in vitro* study has not been evaluated, and energy absorbing factors using techniques such as Fourier-transformed infrared spectroscopy [ 14 ] has not been investigated. In addition, the possible effects of laser irradiation on the periodontal ligament have not been considered. Therefore, further studies are required before Er: YAG laser can be verified as a bond-reducing removal method in clinical settings.

## Conclusion

According to the results of the present study, the most effective Er: YAG laser parameters for reducing bond strength was irradiation for 1 min, at 7 W, 350 mJ and frequency of 20 Hz, and discontinued washing sprays. Using these parameters, the findings showed that while complete debonding was not achieved, Er: YAG could effectively reduce bonding integrity and tensile resistance of glass fiber posts. These findings highlight the potential effect of Er: YAG laser irradiation in facilitating the removal of glass fiber posts cemented in root canaled premolar teeth with resin cement.

## References

[1] Cheung W ( 2005). A review of the management of endodontically treated teeth: Post, core and the final restoration. J Am Dent Assoc.

[2] ParčinaAmižić I, Baraba A ( 2016). Esthetic intracanal posts. Acta Stomatolcroat.

[3] Maccari PC, Conceicao EN, Nunes MF ( 2003). Fracture resistance of endodontically treated teeth restored with three different prefabricated esthetic posts. J Esthet Restor Dent.

[4] Goracci C, Ferrari M ( 2011). Current perspectives on post sy-stems: a literature review. Aust Dent J.

[5] Morgano SM, Rodrigues AH, Sabrosa CE ( 2004). Restoration of endodontically treated teeth. Dent Clin North Am.

[6] Abbott P ( 2002). Incidence of root fractures and methods used for post removal. Int Endontic J.

[7] Castrisos T, Abbott P ( 2002). A survey of methods used for post removal in specialist endodontic practice. Int Endontic J.

[8] Pithon MM, Figueiredo DSF, Oliveira DD, da Silva Coqueiro R (2015). What is the best method for debonding metallic brackets from the patient’s perspective?. Prog Orthod.

[9] Walsh L ( 2003). The current status of laser applications in dentistry. Aust Dent J.

[10] Stabholz A, Zeltser R, Sela M, Peretz B, Moshonov J, Ziskind D ( 2003). The use of lasers in dentistry: principles of operation and clinical applications. Compend Contin Educ Dent.

[11] Bader C, Krejci I ( 2006). Indications and limitations of Er: YAG laser applications in dentistry. Am J Dent.

[12] Oztoprak MO, Nalbantgil D, Erdem AS, Tozlu M, Arun T ( 2010). Debonding of ceramic brackets by a new scanning laser method. Am J Orthod Dentofacial Orthop.

[13] Mundethu AR, Gutknecht N, Franzen R ( 2014). Rapid debonding of polycrystalline ceramic orthodontic brackets with an Er: YAG laser: an in vitro study. Lasers Med Sci.

[14] Morford CK, Buu NC, Rechmann BM, Finzen FC, Sharma AB, Rechmann P ( 2011). Er: YAG laser debonding of porcelain veneers. LSM.

[15] Pereira JR, do Valle AL, Ghizoni JS, Lorenzoni FC, Ramos MB, dos Reis Só MV ( 2013). Push-out bond strengths of different dental cements used to cement glass fiber posts. J Prosthet Dent.

[16] Hoteit M, Nammour S, Zeinoun T ( 2020). Evaluation of Enamel Topography after Debonding Orthodontic Ceramic Brackets by Different Er,Cr:YSGG and Er:YAG Lasers Settings. Dent J.

[17] Pelozo L, Silva-Neto R, Corona S, Palma-Dibb R, Souza-Gabriel A ( 2019). Dentin pretreatment with Er:YAG laser and sodium ascorbate to improve the bond strength of glass fiber post. Lasers Med Sci.

[18] Gomes KGF, Faria NS, Neto WR, Colucci V, Gomes EA ( 2018). Influence of laser irradiation on the push-out bond strength between a glass fiber post and root dentin. J Prosthet Dent.

[19] Deeb JG, Grzech-Leśniak K, Weaver C, Matys J, Bencharit S ( 2019). Retrieval of glass fiber post using Er:YAG laser and conventional endodontic ultrasonic method: An in vitro study. J Prosthodont.

[20] Apel C, Franzen R, Meister J, Sarrafzadegan H, Thelen S, Gutknecht N ( 2002). Influence of the pulse duration of an Er: YAG laser system on the ablation threshold of dental enamel. J Lasers Med Sci.

[21] Tak O, Sari T, ArslanMalkoç M, Altintas S, Usumez A, Gutknecht N ( 2015). The effect of transmitted Er: YAG laser energy through a dental ceramic on different types of resin cements. LSM.

[22] Yearn J ( 1985). Factors affecting cure of visible light activated composites. Int Dent J.

[23] Ghavam M, Kermanshah H, Ataei M, Shadman N ( 2007). Polymerization of dual cure resin cements applied for luting tooth colored fiber posts. Journal of Dental Medicine.

[24] Onay E, Korkmaz Y, Kiremitci A ( 2010). Effect of adhesive system type and root region on the push‐out bond strength of glass–fibre posts to radicular dentine. Int Endodontic J.

[25] Alexander R, Xie J, Fried D ( 2002). Selective removal of residual composite from dental enamel surfaces using the third harmonic of a Q‐switched Nd: YAG laser. LSM.

[26] Takashina M, Ebihara A, Sunakawa M, Anjo T, Takeda A, Suda H ( 2002). The possibility of dowel removal by pulsed Nd: YAG laser irradiation. LSM.

[27] Angeloni V, Mazzoni A, Marchesi G, Cadenaro M, Comba A, Maravić T, et al ( 2017). Role of Chlorhexidine on Long-term Bond Strength of Self-adhesive Composite Cements to Intraradicular Dentin. J Adhes Dent.

[28] Durski M, Metz M, Crim G, Hass S, Mazur R, Vieira S ( 2018). Effect of Chlorhexidine Treatment Prior to Fiber Post Cementation on Long-Term Resin Cement Bond Strength. Oper Dent.

[29] Oliviera LV, Maia TS, Zancope K, Menezes MDS, Soares CJ, Moura CCG ( 2018). Can intra-radicular cleaning protocols increase the retention of fiberglass posts? A systematic review. Braz Oral Res.

[30] Khalighinejad N, Feiz A, Faghihian R, Swift JE ( 2014). Effect of dentin conditioning on bond strength of fiber posts and dentin morphology: a review. Am J Dent.

[31] Almulhim KS, Oliveira-Haas L, Farhangpour A ( 2016). Effect of resin cement, aging process, and root level on the bond strength of fiber-posts: An in vitro study. Am J Dent.

[32] Ruiz L, Gomes GM, Bittencourt B, da Silva FR, Gomes OMM, ChidoskiFilho JC, et al ( 2018). Effect of Root Canal Sealers on Bond Strength of Fiber Posts to Root Dentin Cemented after one Week or six Months. IEJ.

[33] Marchesi G, Mazzoni A, Turco G, Cadenaro M, Ferrari M, Di Lenarda R, et al ( 2013). Aging affects the adhesive interface of posts luted with self-adhesive cements: a 1-year study. J Adhes Dent.

[34] Kececi AD, Kaya BU, Adanir N ( 2008). Micro push-out bond strengths of four fiber-reinforced composite post systems and 2 luting materials. Oral Surg Oral Med Oral Pathol Oral Radiol Endod.

